# Construction of high‐density genetic linkage map and mapping quantitative trait loci (QTL) for flowering time in autotetraploid alfalfa (*Medicago sativa* L.) using genotyping by sequencing

**DOI:** 10.1002/tpg2.20045

**Published:** 2020-09-26

**Authors:** Fan Zhang, Junmei Kang, Ruicai Long, Long‐Xi Yu, Yan Sun, Zhen Wang, Zhongxiang Zhao, Tiejun Zhang, Qingchuan Yang

**Affiliations:** ^1^ Institute of Animal Science Chinese Academy of Agricultural Sciences Beijing China; ^2^ Plant Germplasm Introduction and Testing Research United States Department of Agriculture‐Agricultural Research Service Prosser WA USA; ^3^ Grassland Science Department, College of Animal Science and Technology China Agricultural University Beijing China; ^4^ Cangzhou Academy of Agriculture and Forestry Sciences Cangzhou China

## Abstract

Flowering time is an important agronomic trait of alfalfa (*Medicago sativa* L.). Managing flowering time can produce economic benefits for farmers. To understand the genetic basis of this trait, quantitative trait loci (QTL) mapping was conducted in a full‐sib population that consisted of 392 individuals segregating based on flowering time. High density linkage maps were constructed using single nucleotide polymorphism (SNP) markers generated by genotyping‐by‐sequencing (GBS). The linkage maps contained 3,818 SNP markers on 64 linkage groups in two parents. The average marker density was 4.33 cM for Parent 1 (P1) and 1.47 cM for Parent 2 (P2). Phenotypic data for flowering time was collected for three years at one location. Twenty‐eight QTLs were identified associated with flowering time. Eleven QTLs explained more than 10% of the phenotypic variation. Among them, five main effect QTLs located on linkage group (LG) 7D of P1 and five main effect QTLs located on LG 6D of P2 were identified. Three QTLs were co‐located with other QTLs. The identified linked markers to QTLs could be used for marker‐assisted selection in breeding programs to develop new alfalfa varieties to modulate flowering time.

## 1 INTRODUCTION

Alfalfa (*Medicago sativa* L.) is an outcrossing autotetraploid species, and is the most important forage legume in the world (May, 2004). Flowering time is one of the most important traits for yield production of alfalfa, and managing flowering time to avoid rainy season harvesting can reduce unnecessary yield losses (Jung & Müller, 2009). Information on flowering time can help farmers choose the most suitable grazing periods and harvest dates, thus maximizing economic benefits (Arzani et al., 2004). Flowering time is a quantitative trait, and is determined by genetic and environmental factors (Koornneef, Alonso‐Blanco, Peeters, & Soppe, 1998). Day‐length and temperature are the two main environmental factors influencing flowering (Bhatt, 1977). Depending on the photoperiod sensitivity, plants can be classified into long‐day, day‐neutral, and short–day groups (Kikuchi & Handa, 2009). Alfalfa is a perennial long‐day plant and its flowering is induced when the photoperiod exceeds the critical threshold value (Bula & Massengale, 1972). As a consequence, the time to flowering in alfalfa decreases as the photoperiod lengthens (Major, Hanna, & Beasley, 1991). Flowering time is also affected by temperature. Lower accumulated temperature values have been shown to delay flowering time, and cold conditions require more time to flowering for alfalfa than warm conditions (Nelson & Smith, 1969).

The genetic mechanism of flowering time has been studied in the model legume *Medicago truncatula* (Weller & Macknight, 2018). Vernalization and long days can promote an extended flowering time (Clarkson & Russell, 1975). A significant number of genes have been validated as controlling flowering time, such as *FTa*, *FTb*, and *FTc* (Hecht et al., 2005). QTL mapping was conducted to identify flowering related QTLs in *Medicago truncatula*. Julier et al. (2007) found a stable QTL on chromosome 7 using a recombinant inbred progeny that explained the majority of the variation. Additionally, Pierre, Bogard, Herrmann, Huyghe, and Julier, (2011) used a fine mapping method to find a major flowering time QTL on chromosome 7. This interval contained *FT‐like* and *CO‐like* genes. As for alfalfa, association mapping of flowering time using a candidate gene approach has been carried out previously (Herrmann, Barre, Santoni, & Julier, 2010). The result showed that a *CONSTANS‐LIKE* gene was related to flowering time in alfalfa. Additionally, the activity of several known functional genes involved in flowering, such as *MsZFN* (Chao et al., 2014), *MicroRNA156* (Aung et al., 2015) and *MsLFY* (Zhang, Chao, Kang, Ding, & Yang, 2013), have been reported in alfalfa. However, little is known regarding the genetic architecture of alfalfa flowering time. Genotyping‐by‐sequencing (GBS) (Elshire et al., 2011) is a useful technology for sequencing plants with a lack of a reference genome. In alfalfa, GBS has been used to construct a high‐density linkage map (Adhikari, Lindstrom, Markham, & Missaoui, 2018, Li et al., 2014). Furthermore, genome‐wide association studies (GWAS) have been applied to mapping loci associated with drought and salt tolerances in a diverse panel of alfalfa germplasm using GBS (Meng, Li, Zhang, & Wang, 2015; Yu, Liu, Boge, & Liu, 2016).

**Core Ideas**

- A full‐sib F1 population segregating on flowering time were developed and phenotyped.
- Genotyping‐by‐sequencing was used for genotyping individuals.
- High density linkage maps were constructed using single nucleotide polymorphism (SNP) markers.
- Twenty‐eight QTLs were identified to be associated with flowering time.
- Five major QTLs were located on linkage group 7D of P1.

In the present study, we evaluated a full‐sib population consisting of 392 progeny lines segregating for flowering time in field trials over a 3‐year period. We applied QTL mapping for the identification of loci associated with flowering time. Our ultimate goal was to understand the genetic basis of flowering time in alfalfa. We aim to identify molecular markers closely linked to the flowering time for marker‐assisted selection in alfalfa breeding programs.

## 2 MATERIALS AND METHODS

### 2.1 Plant materials

**FIGURE 1:**
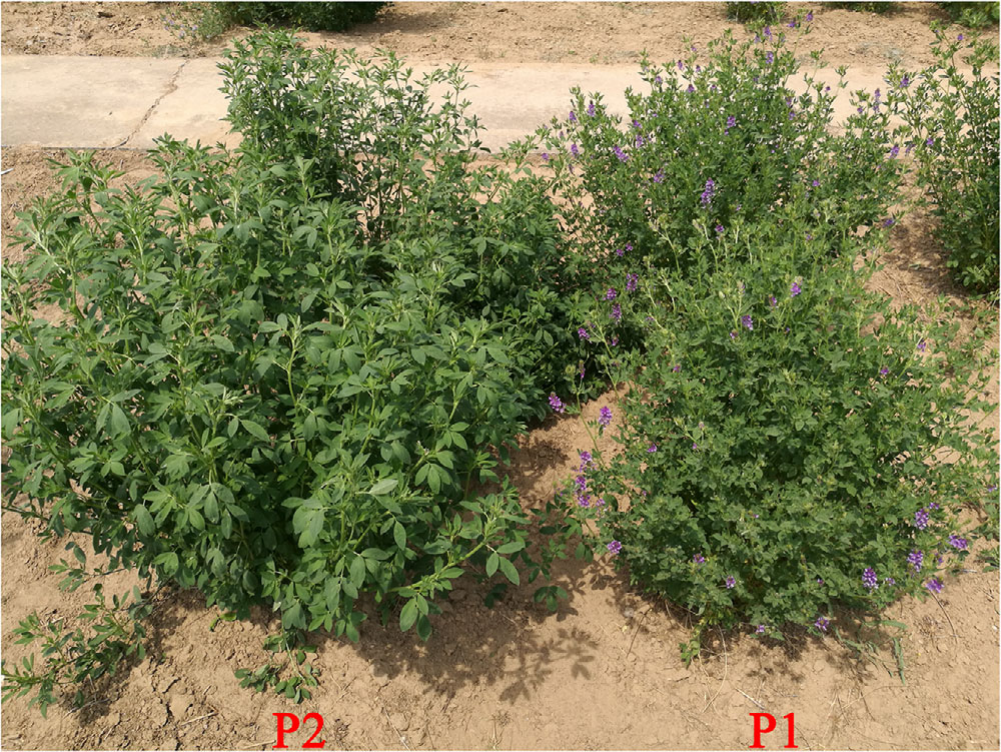
The phenotypic difference between the parents P2 (left) and P1 (right) for flowering time. Flowering time difference are clearly visible on the two parents

The two parents were P1 derived from a local cultivar Cangzhou (CF000735) and P2 derived from cultivar Zhongmu NO.1 (CF0032020). They were crossed to generate an F_1_ population consisting of 392 progenies. P1 (paternal parent) and P2 (maternal parent) were individuals selected from a panel of early flowering and late flowering breeding lines, respectively. The flowering time difference is significant between two parents (Figure 1). In 2015, the seeds of the F_1_ population were planted in the greenhouse of the Chinese Academy of Agricultural Sciences (CAAS) in Langfang (39.59° N, 116.59° E), Hebei Province, China, under conditions of 16 h day/8 h night, 22 °C, and 40% relative humidity. Light intensity (a combination of natural light and light from incandescent lamps) was approximately 200 μmol m^−2^ s^−1^. Clones were propagated from each individual by stem cuttings. During the early branching stage (12 Apr. 2016), the cloned plants were removed from propagation flats and transplanted to the field in the research station of CAAS in Langfang, Hebei Province. The field trial was carried out using a randomized complete block design with three replicates. Every replicate contained 392 individuals with one cloned plant. The plant spacing was 100 cm between rows and 80 cm within rows. The block size was 320 m^2^, and three blocks had the same size. When plants begin to flower, the plants were clipped to a height of 5 cm. To ensure the plants were uniform, they were clipped twice before phenotyping.

### 2.2 Phenotyping

Flowering time (the date at which the first flower appeared) was measured for the first cycle (every day from May to June), in 2016, 2017, and 2018 in Langfang, and adjusted to photothermal units. We converted the flowering time date to photothermal units (PTU) using the method described by Grabowski et al. (2017). Average daily temperature (avgT) was calculated as: avgT = (minT + maxT)/2. Where minT was the minimum recorded temperature and maxT was the maximum recorded temperature. In the PTU equation, Days were defined as the sum days from the date accumulating after 5 consecutive days with avgT > 10 °C to the date the first flower appeared; dayL was defined as the number of hours between sunrise and sunset. The information of avgT and dayL were collected using a weather station at Yi Kang Nong (Beijing, China). A normality test of means of three years PTU was conducted using SAS PROC UNIVARIATE (SAS Institute). The ANOVA and interaction of genotype with years was conducted using PROC GLM (SAS Institute, Cary, NC, USA, 2010). The random effect model as follows:

$$\mathrm{PT}U_{\mathrm{ijk}}=m+y_{k}+r_{i(k)}+g_{j}+gy_{jk}+e_{ijk}$$

in which PTU*ijk* is the PTU data for the *j*th genotype in the *i* replication of the *k*th year. m is the grand mean, y*k* is the kth year, r*i(k)* is the effect of ith replication nested in the *k*th year, g*j* is the genetic effect of the *j*th genotype, gy*jk* is the interaction effect of *j*th genotype and *k*th year, and e*ijk* is the residual. All factors were considered as random.

The broad sense heritability (H^2^) was calculated using the function of AOV of QTL IciMapping (Meng et al., 2015).

### 2.3 DNA extraction, GBS library preparation, and sequencing

DNA was extracted from 100 mg of fresh young leaf tissue using the CWBIO Plant Genomic DNA Kit (CoWin Biosciences, Beijing, China), according to the manufacturer's protocol. DNA was quantified using a Nanodrop 2000 spectrophotometer at an absorbance of 260 nm (Thermo Scientific, Waltham, MA, USA). DNA concentrations were adjusted to 50 ng/μl and subsequently used for GBS library preparation. DNA was digested using the EcoT221 restriction enzyme, and libraries were sequenced on a Hi‐seq2000 (Illumina) line, using 100‐base paired‐end sequencing at the Cornell University Sequencing Facility (Ithaca, NY, USA).

### 2.4 Genotype calling

The Tassel 3.0 Universal Network Enabled Analysis Kit (UNEAK) pipeline (Lu et al., 2013) was used for SNP calling and genotyping. Briefly, the quality check of initial sequences was performed using FastQC (v0.11.5, http://www.bioinformatics.babraham.ac.uk/projects/fastqc/). The FASTQ files were recorded into the tagCount file using UFastqToTagCountPlugin. Each tagCount file was then merged into a single “merged” tagCount file using UMergeTaxaTagCountPlugin with a minimum count tag of five. The merged tagCount file was used for mismatched tag pair alignment using UTagCountToTagPairPlugin with a default error tolerance of 0.03. Next, the UTagPairToTBTPlugin was used to tag distributions in all of the taxa. The UTBTToMapInfoPlugin was used to sort the tagsByTaxa file according to the order of tags recorded in the tagPair file, and was then used to convert each tag pair to a HapMap record and assign genotypes to each taxa. Finally, HapMap files were generated using UMapInfoToHapMapPlugin with the MAF value from 0.05 to 0.5. The raw data of GBS were submitted to the NCBI Sequence Read Archive with bioproject ID: PRJNA522887 (https://www.ncbi.nlm.nih.gov/bioproject/?term=PRJNA522887).

### 2.5 Genetic linkage map construction and QTL mapping

Single‐dose alleles (SDA; AAAB × AAAA) were calculated according to the method of Li et al. (2014). Briefly, the SDA with a segregation ratio of less than 2:1 in F1 progenies were consider as SDA markers, while those SDAs with fewer than 50% missing values among F1 progeny were used for linkage group construction using JoinMap. Initially, in order to group 32 linkage groups, SDA markers were grouped using a minimum logarithm of odds (LOD) score of 7 for P1 and 16 for P2. Following this, markers were ordered using the regression algorithm with the minimum LOD score of 1 and maximum recombination frequency of 0.4. The map distance was calculated using the function of Kosambi. The R package R/qtl was used for display of the linkage map (Arends, Prins, Jansen, & Broman, 2010). Based on the BLAST information of SNP markers, each four linkage groups were assigned to a corresponding chromosome of *Medicago truncatula*. QTL IciMapping was used to identified QTLs with the functionality of BIP by inclusive composite interval mapping with an additive effect (ICIM‐ADD) (Meng et al., 2015). A LOD threshold of 3 was used for declaring significant QTL.

## 3 RESULTS

### 3.1 Phenotypic analysis

The average PTU values for the trials in 2016, 2017, and 2018 were 186.11, 218.78, and 209.88, respectively, while the coefficients of variation during the same years were 16.5%, 15.0%, and 18.7%, respectively (Supplemental Table S1). The broad‐sense heritabilities (*H* ^2^) in the three years were 86.0%, 85.2% and 85.2% (Table 1). The *P* values of the Kolmogorov–Smirnov test for normality were lower than the threshold (0.05) in the three years, indicating the hypothesis of a non‐normal distribution of PTU in the F_1_ populations was correct. Significant differences of genotype were found in each year among the 392 F_1_ individuals. Additionally, the genotype × year interaction was significant (Table 2). Single year PTU (2016, 2017, and 2018) values were used for QTL mapping.

**TABLE 1:** Summary statistics of photothermal units (PTU) of flowering time in the F1 population in 2016, 2017, and 2018

Year	n	Mean	Range	CV (%)	Kurtosis	Skewness	*P* value (D test)	*H* ^2^ (%)
2016	392	186.1	140.2–273.4	16.5	−0.9	0.4	<0.01	86.0
2017	392	218.9	151.1–317.3	15.0	−0.7	0.2	<0.01	85.2
2018	392	210.1	132.2–307.7	18.7	−0.8	0.1	<0.01	85.2

CV, coefficient of variation. D test, Kolmogorov–Smirnov test. H^2^, broad‐sense heritability based on individual mean.

**TABLE 2:** Variance analysis of photothermal units (PTU) of flowering time in a F1 population in 3 years using mixed model

	df	Type III SS	F value	*P* value
Genotype	391	2704511.5	16.8	<.0001
Year	2	574875.0	698.2	<.0001
Replication	2	22747.3	27.6	<.0001
Genotype × Year	740	424643.8	1.4	<.0001

### 3.2 Genetic linkage map construction

**FIGURE 2:**
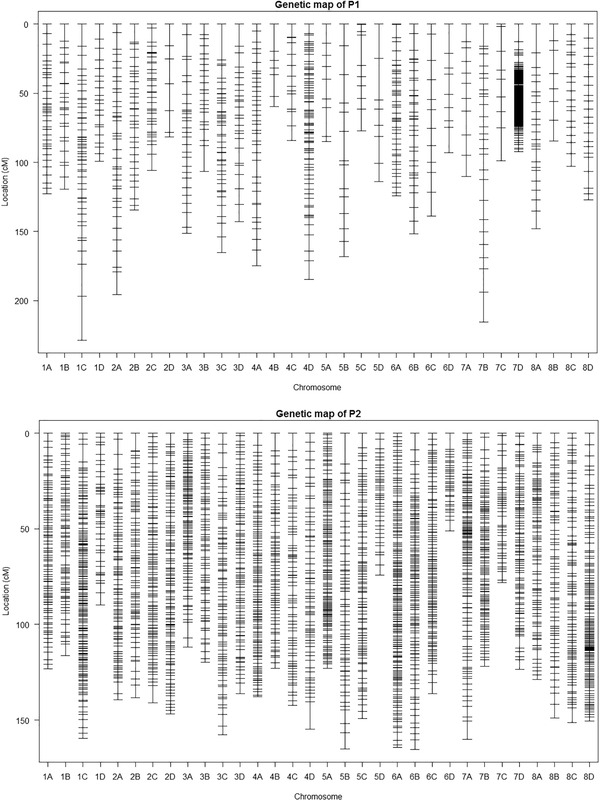
Genetic linkage maps of P1 and P2. The location indicates the positions of markers in Kosambi centiMogan (cM). The chromosome indicates 32 linkage groups. Each linkage group is named based on *Medicago truncatula*, with four LGs (A to D) on each of eight chromosomes

A total of 66,250 SNP markers were generated using the universal network enable analysis kit (UNEAK) pipeline (Lu et al., 2013). A total of 28,684 SNP markers with less than 50% missing genotypic value were used for single dose alleles (SDA) selection. SDA were 1,510 for P1 and 3,883 for P2. For each parent, SDA markers were grouped into 32 linkage groups (LG) using JoinMap (Table 3). The P1 parent spanned a total length of 4,088.70 cM, with 944 mapped SNP markers and an average marker density of 4.33 cM. For P1, the length of LG ranged from 59.68 cM (LG 4B) to 228.53 cM (LG 1C). There were 7 (LG 4B) to 186 (LG 7D) markers distributed on different LGs (Table 3 and Figure 2). The P2 linkage map included 2,874 SNP markers and covered a total of 4,229.15 cM, with an average density of 1.5 cM per marker. The LGs contained markers from 31 (LG 6D) to 188 (LG 1C). The length of LG ranged from 51.15 cM (LG 6D) to 165.27 cM (LG 6B) (Table 3 and Figure 2).

**TABLE 3:** Distribution of SNP markers among 32 linkage groups of P1 and P2 parents of the F1 mapping population and the length of each haplotype map

		P1		P2	
Chromosome	Group	Marker, No.	Length, cM	Marker, No.	Length, cM
1	A	37	122.76	81	123.28
1	B	27	119.37	82	116.32
1	C	49	228.53	188	159.53
1	D	21	99.31	40	89.85
2	A	37	195.74	85	139.40
2	B	33	134.62	80	138.31
2	C	36	105.86	96	140.85
2	D	8	81.72	102	146.88
3	A	28	151.33	99	111.92
3	B	22	106.63	68	119.83
3	C	41	165.33	81	157.69
3	D	26	142.98	88	136.17
4	A	37	174.81	106	137.74
4	B	7	59.68	70	122.96
4	C	14	84.12	69	142.24
4	D	75	184.49	64	154.70
5	A	9	85.00	143	122.82
5	B	14	168.32	58	165.12
5	C	10	77.41	86	149.21
5	D	8	114.06	37	74.07
6	A	44	124.17	178	164.23
6	B	33	151.79	107	165.27
6	C	12	138.73	102	136.32
6	D	9	93.13	31	51.15
7	A	11	110.11	127	160.15
7	B	28	215.36	108	122.01
7	C	9	98.85	41	78.12
7	D	186	92.16	86	123.53
8	A	24	147.83	85	128.74
8	B	8	84.53	70	148.84
8	C	20	102.96	83	151.44
8	D	21	127.01	133	150.46
Total		944	4088.70	2874	4229.15
average			4.33		1.47

### 3.3 QTL mapping of flowering time

**FIGURE 3:**
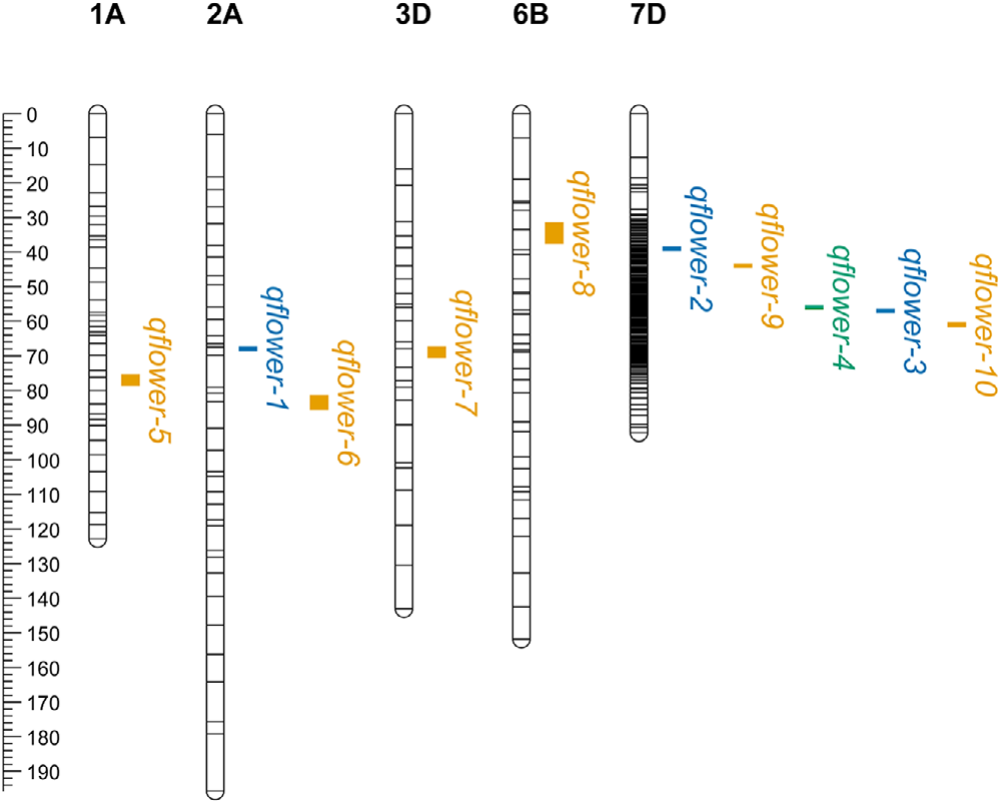
Flowering time related QTLs on 32 linkage groups from a genetic linkage map of parent 1 (P1). Distances among markers are indicated in cM to the left ruler of the figure. QTLs are depicted as colored vertical bars to the right of the linkage groups. Different colors represent different years. The color of QTL (blue, blue‐green, and orange) represent the years 2016, 2017, and 2018. Groups with no detected QTLs are not shown

**FIGURE 4:**
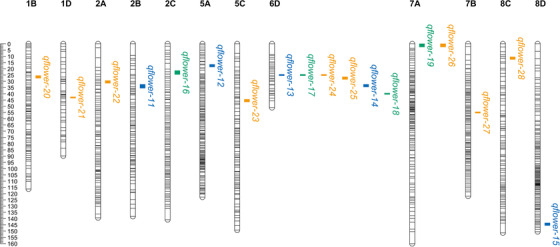
Flowering time related QTL on 32 linkage groups from a genetic linkage map of parent 2 (P2). Distances among markers are indicated in cM to the left ruler of the figure. QTLs are depicted as colored vertical bars to the right of the linkage groups. Different colors represent different years. The color of QTL (blue, blue‐green, and orange) represent the years 2016, 2017, and 2018. Groups with no detected QTLs are not shown

A total of 28 QTLs were identified in P1 and P2 among the three year test groups. The percentage of phenotypic variation explained by these QTLs varied from 2.1% to 59.6% (Table 4). There were 11 main effect QTLs that explained more than 10% of the phenotypic variance (PVE) for flowering time. Five main effect QTLs (*qflower‐2*, *qflower‐3*, *qflower‐4*, *qflower‐9*, and *qflower‐10*) were located on LG 7D of P1 and five main effect QTLs (*qflower‐13*, *qflower‐17*, *qflower‐18*, *qflower‐24*, and *qflower‐25*) were located on 6D of P2. Another main effect QTL (*qflower‐1*) was located on LG 2A at the position from 67.5 cM to 68.5cM of P1. The five main effect QTLs located on LG 7D of P1 were at the position from 38.5 cM to 61.5 cM (23 cM interval). The five main effect QTLs located on LG 6D of P2 were at the position from 24.5 cM to 40.5 cM (16 cM interval). Three co‐located (QTLs locate in the same interval) QTLs were observed in P1 and P2 (Figure 3 and Figure 4). The QTL *qflower‐17* was detected on LG 6D of P2. This QTL had the highest LOD value of 60.7 and explained 53.1% of the phenotypic variance, and was co‐located with *qflower‐13* and *qflower‐24*. The QTL *qflower‐4* was detected on LG 7D of P1. It had the highest PVE value of 59.6 and was co‐located with *qflower‐3*. The QTL *qflower‐19* was co‐located with *qflower‐26*, and was located on LG 7A of P2.

**TABLE 4:** Quantitative trait loci (QTL) associated with flowering time identified by inclusive composite interval mapping of additive effects

Parent	Year	QTL	LG	Position	Left Marker	Right Marker	LOD interval (cM)	LOD	PVE(%)	Add
P1	2016	*qflower‐1*	2A	68.0	TP31403	TP3688	67.5–68.5	4.8	12.2	5.0
P1	2016	*qflower‐2*	7D	39.0	TP84873	TP21323	38.5–39.5	7.5	15.8	−6.0
P1	2016	*qflower‐3*	7D	57.0	TP55339	TP53717	56.5–57.5	13.3	32.5	−8.2
P1	2017	*qflower‐4*	7D	56.0	TP11147	TP46531	55.5–56.5	14.1	59.6	−8.4
P1	2018	*qflower‐5*	1A	76.0	TP7452	TP64414	75.5–78.5	4.2	6.9	5.6
P1	2018	*qflower‐6*	2A	83.0	TP61240	TP34664	81.5–85.5	4.3	7.4	5.9
P1	2018	*qflower‐7*	3D	68.0	TP68619	TP88414	67.5–70.5	3.4	5.2	−5.0
P1	2018	*qflower‐8*	6B	34.0	TP50878	TP77253	31.5–37.5	4.3	7.0	5.8
P1	2018	*qflower‐9*	7D	44.0	TP93438	TP62238	43.5–44.5	10.7	17.5	−9.1
P1	2018	*qflower‐10*	7D	61.0	TP53243	TP59776	60.5–61.5	12.2	22.9	−10.1
P2	2016	*qflower‐11*	2B	34.0	TP8180	TP89779	32.5–35.5	3.3	2.2	−3.9
P2	2016	*qflower‐12*	5A	17.0	TP85092	TP100300	16.5–18.5	4.4	2.9	4.4
P2	2016	*qflower‐13*	6D	25.0	TP72418	TP48491	24.5–25.5	55.0	49.8	18.4
P2	2016	*qflower‐14*	6D	33.0	TP8104	TP24250	32.5–34.5	9.7	6.4	6.6
P2	2016	*qflower‐15*	8D	145.0	TP72041	TP97049	143.5–145.5	3.1	2.3	−4.1
P2	2017	*qflower‐16*	2C	23.0	TP35969	TP95447	21.5–24.5	3.4	2.1	−3.8
P2	2017	*qflower‐17*	6D	25.0	TP72418	TP48491	24.5–25.5	60.7	53.1	18.9
P2	2017	*qflower‐18*	6D	40.0	TP87775	TP35294	39.5–40.5	18.0	11.9	9.4
P2	2017	*qflower‐19*	7A	0.0	TP23079	TP33482	0–2.5	4.2	2.5	4.2
P2	2018	*qflower‐20*	1B	26.0	TP14297	TP26155	25.5–27.5	3.2	2.4	−4.8
P2	2018	*qflower‐21*	1D	43.0	TP71973	TP91871	42.5–43.5	4.4	3.0	−5.3
P2	2018	*qflower‐22*	2A	31.0	TP40158	TP50173	29.5–31.5	3.3	2.2	−4.6
P2	2018	*qflower‐23*	5C	46.0	TP13431	TP28432	44.5–46.5	9.4	6.5	−7.9
P2	2018	*qflower‐24*	6D	25.0	TP72418	TP48491	24.5–25.5	20.9	16.3	12.3
P2	2018	*qflower‐25*	6D	27.0	TP53281	TP57492	26.5–28.5	36.0	32.2	17.4
P2	2018	*qflower‐26*	7A	0.0	TP23079	TP33482	0–2.5	4.7	3.1	5.5
P2	2018	*qflower‐27*	7B	55.0	TP6175	TP19745	54.5–55.5	3.7	2.8	−5.3
P2	2018	*qflower‐28*	8C	11.0	TP18263	TP99362	10.5–12.5	4.4	3.8	6.3

Parent, QTLs were identified in P1 or P2 linkage map. Position, the position of QTLs on LG. LeftMarker and RightMarker, left and right marker of the QTLs.

LG, linkage group (LG); LOD interval (cM), 1‐LOD support interval; LOD: the logarithm of the odds; PVE: the percentage of the phenotypic variation explained by QTL; Add: the additive effects of the QTL.

## 4 DISCUSSION

### 4.1 Genetic linkage map construction

Several linkage maps have been constructed using GBS SNP markers (Adhikari et al., 2018; Li et al., 2014). In the current study, we used the method described by Li et al. to construct a linkage map for alfalfa (Li et al., 2014). The P2 parent had similar marker density (1.5 cM) to that seen in Li's study (1.0–1.5 cM). However, the average marker density of the P1 parent was 4.33 cM. The total number of mapped markers on P1 was 944, which was less than the marker number (2,874) of P2. This discrepancy may be due to the genetic differences between the two parents. Great genetic diversity has been identified among individuals and allelic variations were found between cultivars and landraces in autotetraploid alfalfa (Qiang et al., 2015). There are typically many phenotypic differences between two parents used in the present study, such as flowering time, yield, leaf size, and regrowth. Furthermore, most SNP information was filtered out using presence/absence of SDA markers (Hackett, Mclean, & Bryan, 2013). Hackett et al. (2013) found that there were 11 possible genotype configurations, but we limited our study to only two (AAAA × AAAB and ABBB × BBBB). In the SNP data file, we found that three were more polymorphic markers in two parents (5,449) than only in P1 (5,237). These two reasons (genetic difference and SNP filter) may have contributed to the different marker density in P1 and P2.

The total length of the two maps were similar (4,088.7 cM for P1 and 4,229.15 cM for P2), and longer than other linkage maps (Adhikari et al., 2018; Li et al., 2014). The differences in recombination frequency may have caused our map length to be longer than previous reports (Williams, Goodman, & Stuber, 1995). The linkage group of 7D contains more markers than the other groups. It couldn't be divided when using LOD = 30, so we assigned these markers to one group. Nevertheless, compared to previous studies, the density of the linkage map constructed by our study was sufficient for QTL mapping (Mccord et al., 2014; Ray et al., 2015).

### 4.2 QTL mapping

Flowering time is an important trait associated with yield. Our data showed that pearson correlation coefficient between flowering time and biomass yield is −0.66 (*P* < .0001). Flowering time is negative correlated with biomass. Flowering time has been studied in many species, such as switchgrass (Tornqvist et al., 2018), maize (Navarro et al., 2017), and *Medicago truncatula* (Weller & Macknight, 2018). Flowering time related genes have also been reported in alfalfa (Herrmann et al., 2010). The present study mapped QTL for flowering time in a biparental mapping population of tetraploid alfalfa consisted of 392 individuals. Our result suggested that this population was large enough to identify significant QTLs for flowering time. A minimum population size can be calculated using the probability of no target genotype and the frequency of target genotype. Using this population, we identified 11 QTLs that explained high phenotypic variation (PVE > 10%). Most of these QTLs were located on 7D of P1 or 6D of P2. These locations suggest the main effect QTL/gene may be located on a syntenic region. The study by Julier et al. (2007) identified flowering time QTLs on chromosome 1, 3, 5, 7, and 8 of *M. truncatula*, and the stable QTL was located on chromosome 7. In order to keep the nomenclature of our results consistent with Julier et al. (2007), we named the linkage group similarly as that of *M. truncatula*. However, in their study, no flowering time QTLs were found on chromosome 6. The loci we identified on chromosome 6 in this study may be a new flowering time QTL of *M. sativa*. However, future studies will be aimed at establishing whether or not this is in fact a new flowering time QTL.

Three co‐located QTLs were identified in P1 and P2. These QTLs explained a high proportion of phenotypic variation and they were located on LG 6D, 7A, and 7D. The interval of co‐located QTLs was fewer than 2.5 cM, which was similar to previous studies (Adhikari et al., 2018; Li et al., 2015; Mccord et al., 2014). This suggests the linkage map we constructed was suitable for QTL mapping. High heritability of flowering time allowed us to detect these consistent QTLs, and the average PVE was 30.98%. Based on our blast results, several key flowering time related genes (*FT*, *CO* et al.) were located in the interval of QTL qflower‐4 (physical position, chr7: 30393914–34423078). These QTLs contributed major effects to flowering time and may be used for candidate gene identification and marker development in further studies.

### 4.3 QTL implicated for alfalfa breeding

Alfalfa is an outcrossing plant and cultivated alfalfa is highly heterozygous. The genetic differences between subpopulations are large. Identification of good individual plants cannot necessarily be based on morphology (İlhan, Li, Brummer, & Şakiroğlu, 2016). QTL mapping has the potential to identify markers associated with phenotypes. The QTL for flowering time identified in this study could facilitate selection speed and accuracy. The QTL with additive effects could be used to select early flowering or late flowering alfalfa for hybrid breeding. Furthermore, these QTL mapping results could help validate the accuracy of QTLs in other populations. However, the resolution of QTLs obtained in this study is insufficient for marker associated selection. There may be population specific QTLs that are not easily transferred to wider germplasm pools (Sharma et al., 2018). The use of different genetic background populations is needed in order to validate the identified QTLs. Furthermore, association mapping could be used for cross validation (Brachi et al., 2010; Zhao et al., 2007). KASPar is a method that could detect the presence/absence of a marker associated with phenotypic variation in different individuals. It can be used for validating markers linked to flowering time. The validated markers can be used in MAS for flowering time.

## 5 CONCLUSION

A genetic linkage map was constructed using GBS markers. The linkage map contained 3,818 SNP, with 32 linkage groups for each parent. The average marker distances were 4.33 and 1.47 cM, with coverage of 4,088.70 cM and 4,299.15 cM for paternal and maternal linkage maps, respectively. Twenty‐eight QTLs associated with flowering time were identified in the F1 population. Eleven QTLs explained more than 10% of the phenotypic variation. Among them, five main effect QTLs were located on LG 7D of P1 and five main effect QTLs were located on LG 6D of P2. Three QTLs were co‐located with other QTLs, and these were located on LG 7D of P1 and LG 6D and 7A of P2. The average phenotypic variation explained by these co‐located QTLs was 30.98%. These QTLs may play a major role in determining flowering time. We plan to validate these co‐located QTLs in another population in further investigations. After confirmation, valid markers linked to QTLs will be used in marker‐assisted selection and genomic selection in an alfalfa breeding program to accelerate the development of new cultivars.

## AUTHOR CONTRIBUTIONS

QCY and TJZ conceived and designed the experiments. FZ, JMK, RCL, YS, ZW, ZXZ performed the experiments. FZ, TJZ, LXY analyzed the data. FZ, LXY wrote the paper.

## COMPETING INTERESTS

The authors declare that they have no conflict of interest.

## Supporting information

Supplemental Table S1. Phenotypic data of photothermal units (PTU) of 392 F1 individuals and parents in 2016, 2017 and 2018.
